# Supplementary figures and images for: Hemigrapsus sanguineus in Long Island salt marshes: experimental evaluation of the interactions between an invasive crab and resident ecosystem engineers
Source: PeerJ. 2014 Jul 3;2:e472. doi: 10.7717/peerj.472 (PMC4103090; doi:10.7717/peerj.472)

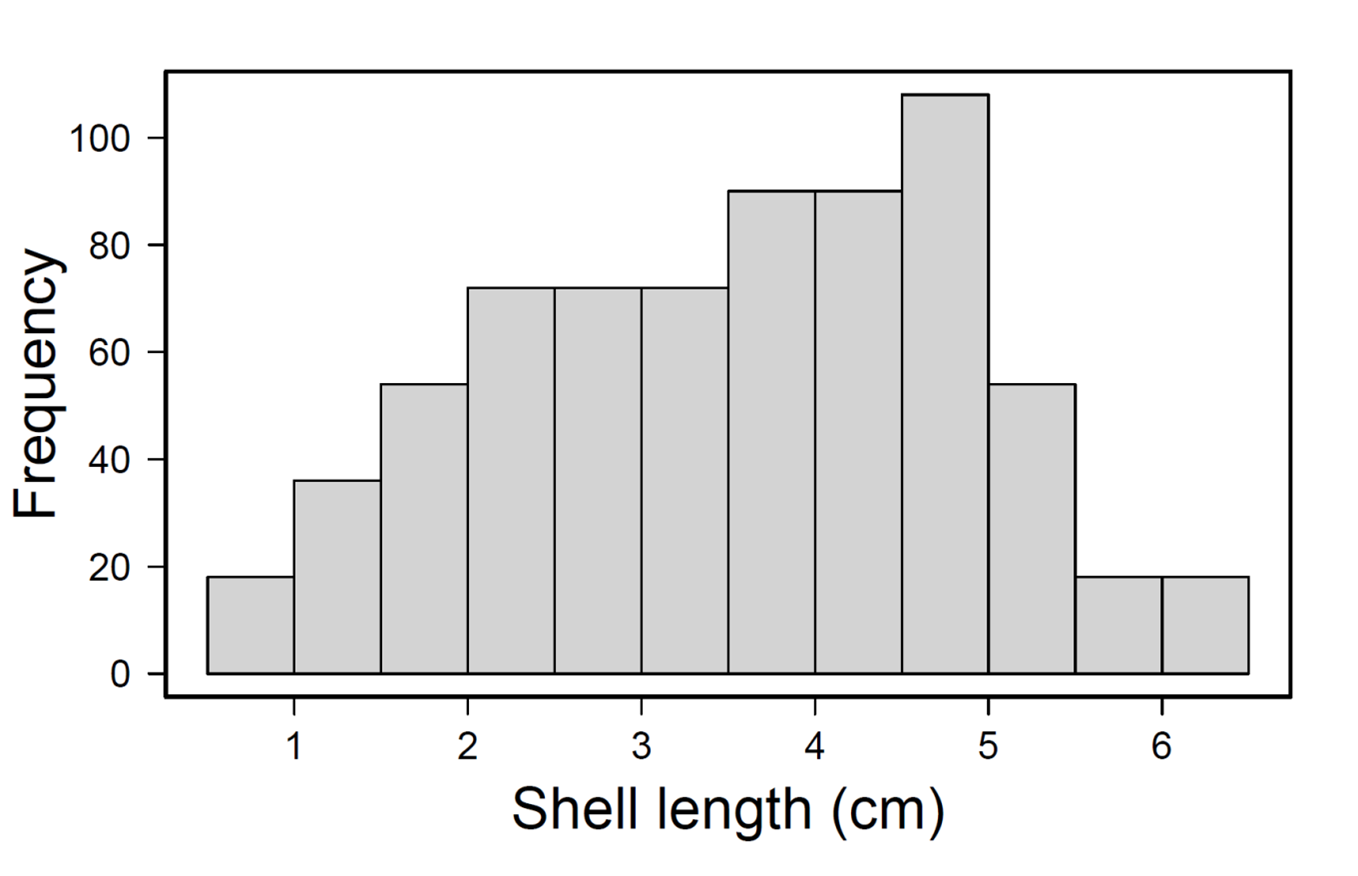

Supplement: Figure S1 — The natural size distribution of the mussels that were used to create the 18 subunits of the same size distributions for the crab predation experiments. [file peerj-02-472-s001.png]
